# Supplementary material for: Escherichia coli Activate Extraintestinal Antibody Response and Provide Anti-Infective Immunity
Source: Int J Mol Sci. 2024 Jul 7;25(13):7450. doi: 10.3390/ijms25137450 (PMC11242715; doi:10.3390/ijms25137450)
Supplement: Supplementary file 1 [file ijms-25-07450-s001.zip › ijms-3081962-supplementary.pdf]

International Journal of
Molecular Sciences

MDPI

Article

Escherichia coli Activate Extraintestinal Antibody Response and Provide Anti-infective Immunity

Xiang Liu, Xuan-xian Peng * and Hui Li *

State Key Laboratory of Bio-Control, School of Life Sciences, Southern Marine Science and Engineering Guangdong Laboratory (Zhuhai), Guangdong Key Laboratory of Pharmaceutical Functional Genes, Sun Yat-sen University, Guangzhou 510275, China.

* Correspondence: pxuanx@sysu.edu.cn (X.X.P.); lihui32@sysu.edu.cn (H.L.); Tel.: +86-13580548832 (X.X.P.); +86-15920516230 (H.L.)

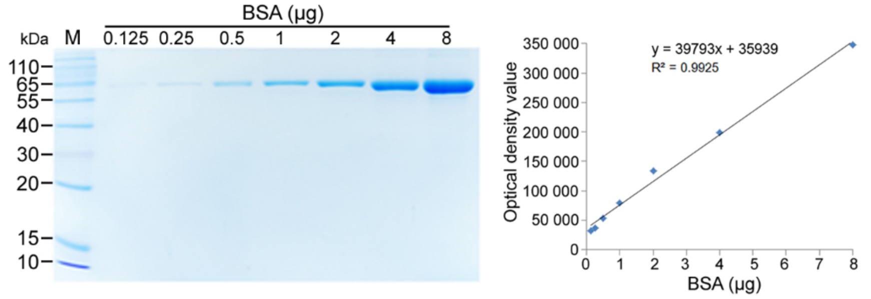

Figure S1. SDS-PAGE analysis for different BAS concentrations as a standard protein concentration.

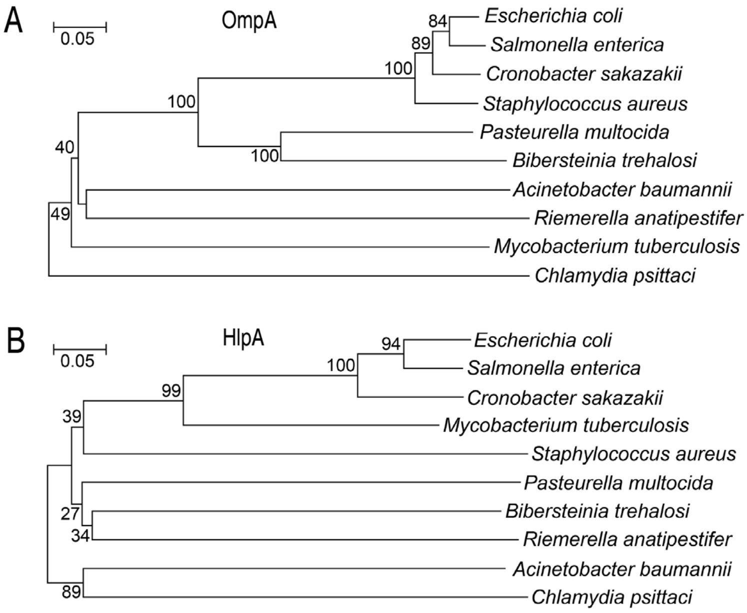

Figure S2. Construction of phylogenetic trees of OmpA and HlpA. A phylogenetic tree with bootstrap values based on the full-length protein sequences of OmpA (A) and HlpA (B). The tree was constructed using a neighbor-joining method.

Citation: Liu, X.; Peng, X.; Li, H. *Escherichia coli* Activate Extraintestinal Antibody Response and Provide Anti-infective Immunity. *Int. J. Mol. Sci.* **2024**, *25*, x. https://doi.org/10.3390/xxxxx

Academic Editor(s): Name

Received: date
Revised: date
Accepted: date
Published: date

Copyright: © 2024 by the authors. Submitted for possible open access publication under the terms and conditions of the Creative Commons Attribution (CC BY) license (https://creativecommons.org/licenses/by/4.0/).

Table S1. Information of 69 OMPs of *E. coli* BW25113 (GenBank: CP009273.1).

GenBank No.	Protein name	Gene name	MW (kDa)
AIN34438.1	Outer membrane lipoprotein	*blC*	18
AIN32568.1	Ferric iron outer membrane transporter	*cirA*	73.9
AIN31061.1	Putative transport	*cusC*	50.3
AIN30722.1	Copper homeostasis protein	*cutF*	25.8
AIN32752.1	Long-chain fatty acid transport protein	*fadL*	48.5
WP_202790439.1	Outer membrane usher protein	*faeD*	85
AIN34564.1	Citrate-dependent iron transport	*fecA*	85
AIN31074.1	Outer membrane receptor for ferric enterobactin	*fepA*	82.1
AIN30680.1	Outer membrane protein receptor for ferrichrome	*fhuA*	82.2
AIN31565.1	Outer membrane receptor for ferric iron uptake	*fhuE*	38
AIN34591.1	Export and assembly of type 1 fimbriae	*fimD*	96.5
AIN31542.1	Flagellar biosynthesis protein	*flgH*	24.6
AIN32418.1	Phase-variable bipartite outer membrane fluffing protein	*flu*	107
AIN30707.1	Periplasmic molecular chaperone for outer membrane proteins	*hlpA*	15.8
AIN33723.1	Putative transport portein	*hofQ*	44.7
AIN30669.1	Probable outer membrane porin protein involved in fimbrial assembly	*htrE*	95.5
AIN34323.1	Phage lambda receptor protein	*lamB*	49.9
AIN31667.1	Lipoprotein localization factor	*lolB*	21
AIN32115.1	Murein sacculus, peptidoglycan	*lpp*	8.3
AIN34367.1	Outer membrane factor of efflux pump	*mdtP*	53
AIN32219.1	Scaffolding protein for murein synthesizing machinery	*mipA*	27.8
AIN33188.1	Membrane-bound lytic murein transglycosylase A	*mltA*	40.4
AIN33076.1	Membrane-bound lytic murein transglycosylase B	*mltB*	40.2
AIN34585.1	N-acetylnuraminic acid outer membrane channel protein	*nanC*	27.9
AIN31057.1	Bacteriophage N4 receptor protein	*nfrA*	111
AIN32878.1	BamABCDE complex OM biogenesis lipoprotein	*nlpB*	36.8
M13457.1	5' IS5 insertion target sequence	*nmpC*	40.3
AIN31425.1	outer membrane protein A	*ompA*	37.2
AIN32626.1	outer membrane protein C	*ompC*	40
AIN31397.1	outer membrane protein F	*ompF*	39.3
AIN31779.1	outer membrane protein G	*ompG*	34.9
AIN31830.1	outer membrane protein N	*ompN*	41.2
TXR28241.1	outer membrane protein P	*ompP*	35
AIN31054.1	outer membrane protein T	*ompT*	35.7
AIN31713.1	outer membrane protein W	*ompW*	22.9
AIN31283.1	outer membrane protein X	*ompX*	18.6
AIN30591.1	LPS assembly OM complex LptDE, beta-barrel component	*ostA*	89.6
AIN31217.1	peptidoglycan-associated outer membrane lipoprotein	*paL*	19
AIN30760.1	Outer membrane phosphoporin protein E	*phoE*	38.9
AIN34131.1	Outer membrane phospholipase A	*pldA*	33
AIN31047.1	Putative lipoprotein, phage or prophage Related	*rzoD*	6.7
AIN33832.1	Outer membrane lipoprotein	*slp*	20.9
AIN32080.1	Outer membrane lipoprotein	*slyB*	15.6
AIN33386.1	Cell division, transport channel	*tolC*	53.7

AIN30910.1	Receptor of phage T6 and colicin K	*tsX*	33.6
AIN32754.1	Lipoprotein precursor	*vacJ*	27
AIN32480.1	Putative polysaccharide export protein	*wzA*	40
AIN30706.1	BamABCDE complex OM biogenesis outer membrane pore-forming assembly factor	*yaeT*	90.6
AIN31194.1	Putative outer membrane protein	*ybgQ*	39.8
AIN31241.1	Acyl-CoA thioesterase, lipoprotein	*ybhC*	46.3
AIN31274.1	Catecholate siderophore receptor	*ybiL*	81.96
AIN31334.1	Putative lipoprotein	*ybjP*	18.99
AIN31408.1	Putative outer membrane fimbrial subunit export usher protein	*ycbS*	95.2
AIN31451.1	Putative O-antigen capsule outer membrane auxillary protein export channel	*yccZ*	41
VWQ02925.1	Eyelet of channel	*yedS*	43
AIN32525.1	Putative outer membrane protein	*yehB*	92.3
AAA16385.1	Pertactin-like passenger domains	*yejo*	91.2
1450268	Outer membrane lipoprotein	*yfcU*	97.5
AIN32913.1	BamABCDE complex OM biogenesis lipoprotein	*yfgL*	41.9
AIN32994.1	OM biogenesis lipoprotein	*yfiO*	27.8
AIN33559.1	Putative outer membrane fimbrial subunit usher protein	*yhcD*	86.3
AIN33911.1	Putative outer membrane protein	*yiaT*	27.4
AIN34045.1	Carbohydrate-specific outer membrane porin	*yieC*	60.7
AAA60501.1	Multidrug resistance outer membrane protein	*yohG*	43
AAA60502	Multidrug resistance outer membrane protein	*yohH*	10.4
AIN33040.1	Putative ATP-binding component of a transport system	*ypjA*	157.5
AIN33366.1	Outer membrane lipoprotein	*yqhH*	9.5
CAD5996707.1	Outer membrane usher protein	*yqiG*	90
AIN33488.1	Putative outer membrane protein	*yraJ*	93

Table S2. Percentage of immunoglobulins bound with *E. coli* in serum immunoglobulin using the standard curve.

	**Binding (IgM--Heavy chain) (μg)**	**Binding (IgG-Heavy chain) (μg)**	**Binding (Light chain) (μg)**	**Total binding (μg)**	**Serum antibody (μg)**	**Binding rate (%)**
a	7.365	7.154	2.81	34.656	57600	0.06
b	5.659	5.647	2.421	27.493	43200	0.064
c	2.987	2.89	1.418	14.591	28800	0.051
d	1.5	1.484	0.761	7.491	14400	0.052
Average	4.378	4.294	1.853	21.058	36000.000	0.057
